# High Current Emission from Patterned Aligned Carbon Nanotubes Fabricated by Plasma-Enhanced Chemical Vapor Deposition

**DOI:** 10.1186/s11671-015-1192-9

**Published:** 2015-12-15

**Authors:** Linfan Cui, Jiangtao Chen, Bingjun Yang, Tifeng Jiao

**Affiliations:** State Key Laboratory of Metastable Materials Science and Technology, Yanshan University, Qinhuangdao, 066004 People’s Republic of China; Hebei Key Laboratory of Applied Chemistry, School of Environmental and Chemical Engineering, Yanshan University, Qinhuangdao, 066004 People’s Republic of China; Laboratory of Clean Energy Chemistry and Materials, State Key Laboratory of Solid Lubrication, Lanzhou Institute of Chemical Physics, Chinese Academy of Sciences, Lanzhou, 730000 People’s Republic of China

**Keywords:** Carbon nanotube, PECVD, Field emission, Emission stability

## Abstract

Vertically, carbon nanotube (CNT) arrays were successfully fabricated on hexagon patterned Si substrates through radio frequency plasma-enhanced chemical vapor deposition using gas mixtures of acetylene (C_2_H_2_) and hydrogen (H_2_) with Fe/Al_2_O_3_ catalysts. The CNTs were found to be graphitized with multi-walled structures. Different H_2_/C_2_H_2_ gas flow rate ratio was used to investigate the effect on CNT growth, and the field emission properties were optimized. The CNT emitters exhibited excellent field emission performance (the turn-on and threshold fields were 2.1 and 2.4 V/μm, respectively). The largest emission current could reach 70 mA/cm^2^. The emission current was stable, and no obvious deterioration was observed during the long-term stability test of 50 h. The results were relevant for practical applications based on CNTs.

## Background

Field emission is a quantum mechanical tunneling phenomenon. Electrons in the materials can emit into vacuum from solid surface which is determined by the strength of local electric field and potential barrier to emission. Field emission occurs from a cold cathode at room temperature which is more power efficient than thermionic emission [1]. Field emission is widely used in many kinds of vacuum electronic applications such as flat panel displays, microwave power tubes, electron sources, and electron-beam lithography. However, high local filed is required to obtain useful current. In order to reduce the extraction voltage, field emitters with sharp protruding microstructures can be used such as Spindt tip cathodes [2, 3], silicon tips [4, 5], and carbon-based materials [6–9]. Carbon nanotube (CNT) has been recognized as an ideal candidate material for field emission applications due to its unique structure and remarkable mechanical, electrical, and chemical stability. Furthermore, the small tip radius and high aspect ratio of CNT can result in electron emission at extraordinary low-threshold electric field and obtain a high-field enhancement factor. Since the first field emission behavior of CNT reported in 1995, many works showed that the CNT emitters exhibited excellent field emission properties [10–15].

The electron emission of CNTs is originated from the tip of the nanotubes because the electrons located at the tips can easily participate in the field emission [16, 17]. Furthermore, the aligned CNTs with uniform length exhibit better field emission properties than random arrangement ones [18]. The CNT arrays can fulfill the requirements for field emission and manipulated as field emission devices directly. Thus, CNTs had better be vertically aligned and oriented toward an anode. Vertically aligned CNTs can be synthesized by chemical vapor deposition methods (CVD). The CVD methods are ideally suited to prepare CNT films on various substrates, and the process can be assisted by microwave of radio frequency plasma [19–22].

As CNTs are capable of emitting efficient high currents, they are potential as emitters in various devices [23, 24]. But nevertheless, the emission densities and short emission lifetimes present obstacles for the practically available electron field emitters based on CNTs. The challenge is to improve the field emission properties of CNTs. It is found that the applied external field is strongly screened when the spacing distance is shorter than the length of the carbon nanotubes [25]. In order to reduce the screen effect, patterning CNT is an efficient method. In this work, the CNT emitters were fabricated using radio frequency plasma-enhanced chemical vapor deposition (PECVD) method on patterned Si substrate. The vertically aligned CNT arrays showed good field emission properties with high emission current and ultra-long-term emission stability which were better than other reported patterned vertical CNTs [26–28].

## Methods

**Fig. 1:**
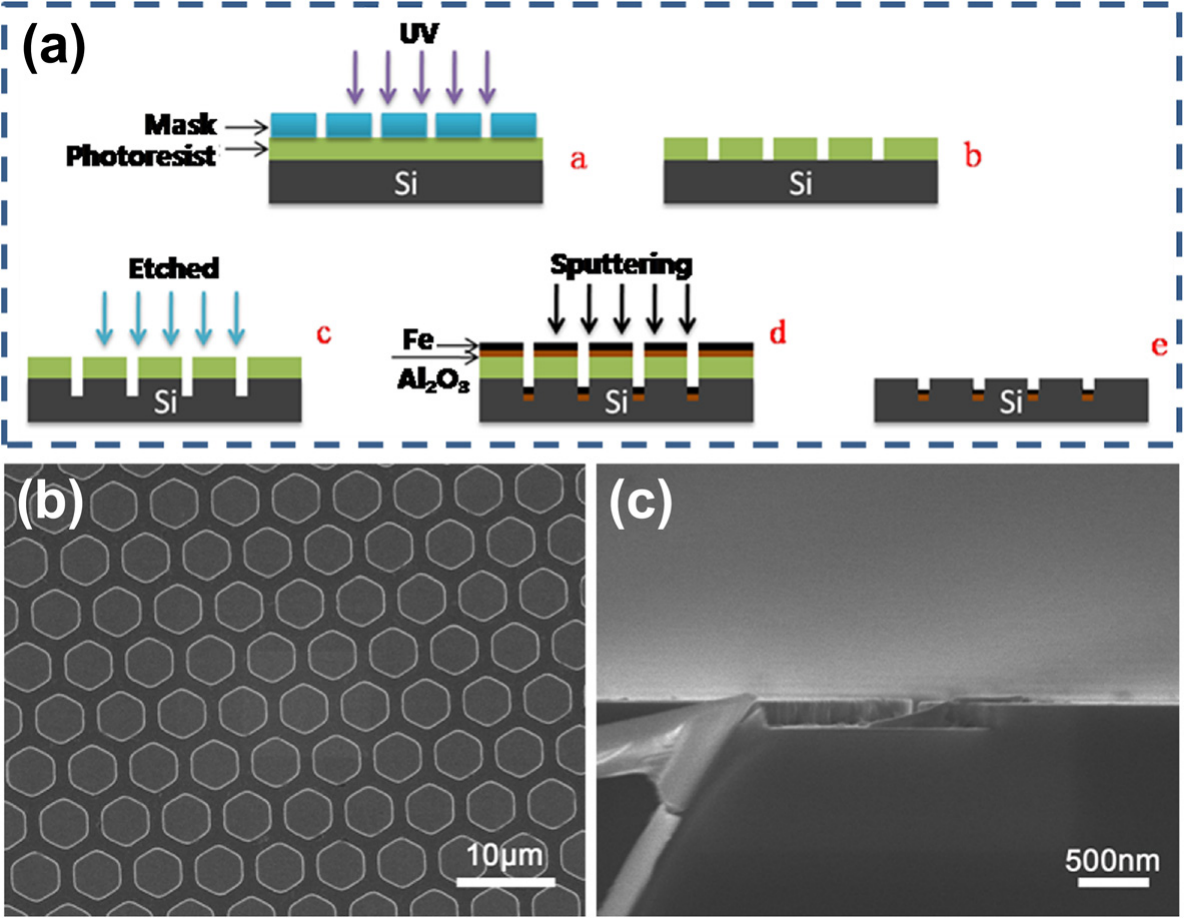
**a** Schematic process of the lithography and catalyst deposition. **b** FESEM images of the patterned Si substrate. **c** Cross-sectional view of the substrate

The prerequisite to make CNTs as electron emitters is to apply them in patterns onto the substrate. Patterned shapes can be accurately prepared and periodically arranged on various substrates by lithography. Figure 1 outlined the lithographic and catalyst deposition process of Si substrate. After ultrasonic cleaning using acetone and alcohol and washing by de-ionized water, low-resistance Si wafers were patterned by a series of techniques (Fig. 1a). Then, Fe thin film with a thickness of 0.8 nm and an Al_2_O_3_ film thickness of 20 nm were deposited on Si substrate by electron beam evaporation. The thickness of the film was controlled by the deposition time. Al_2_O_3_ film was used as an intermediate buffer layer to create roughness and an environment for catalyst nucleation sites. Furthermore, it also could prevent the catalyst from agglomeration effectively [29]. The honeycomb-like patterned catalysts were achieved by removing the photoresist using acetone. Figure 1b showed field emission scanning electronic microscope (FESEM) image of the uniform patterned Si substrate. The side length of each hexagon and the distance between hexagons were 2.5 and 1.5 μm, respectively. The lithography depth of the substrate was about 200 nm as shown in Fig. 1c. In this work, CNTs were prepared by radio frequency PECVD utilizing mixing gases of acetylene (C_2_H_2_) and hydrogen (H_2_). Before the deposition, the vacuum chamber was pumped down to 0.1 Pa. The substrate with catalyst films was heated to 700 °C and processed a pretreatment for 5 min. To be specific, the pretreatment was performed by H_2_ plasma at 200 Pa, the H_2_ gas flow rate was 140 sccm, and the input radio frequency power was 50 W. This process was to remove the oxide catalyst surface and form catalyst nanoparticles acting as a nucleation site for CNT growth. After pretreatment, the CNTs were prepared by H_2_/C_2_H_2_ plasma under the same pressure at 750 °C. Different H_2_/C_2_H_2_ gas flow rate ratio was used to discuss its effect on CNT growth. The gas flow rate was controlled by mass-flow controllers. In each run, the radio frequency power was input at a constant value of 100 W for 10 min.

The surface morphology of samples was observed by FESEM (JSM-6701 F). A transmission electron microscopy (TEM, F-30) with an accelerating voltage of 200 kV was used to characterize the microstructure of CNTs. The microstructure was also investigated by micro-Raman spectroscopy (JY-HR800 spectrometer, the excitation wavelength of 532 nm). The field emission characteristics of the films were measured in a chamber with high vacuum better than 5 × 10^−6^ Pa using a parallel-plate-electrode configuration. The distance between anode and cathode was adjusted to 300 μm using a spiral micrometer. The current-voltage (I–V) characteristics were obtained by LabVIEW program through a Keithley 248 power source with a computer-controlled data-acquisition card.

## Results and Discussion

**Fig. 2:**
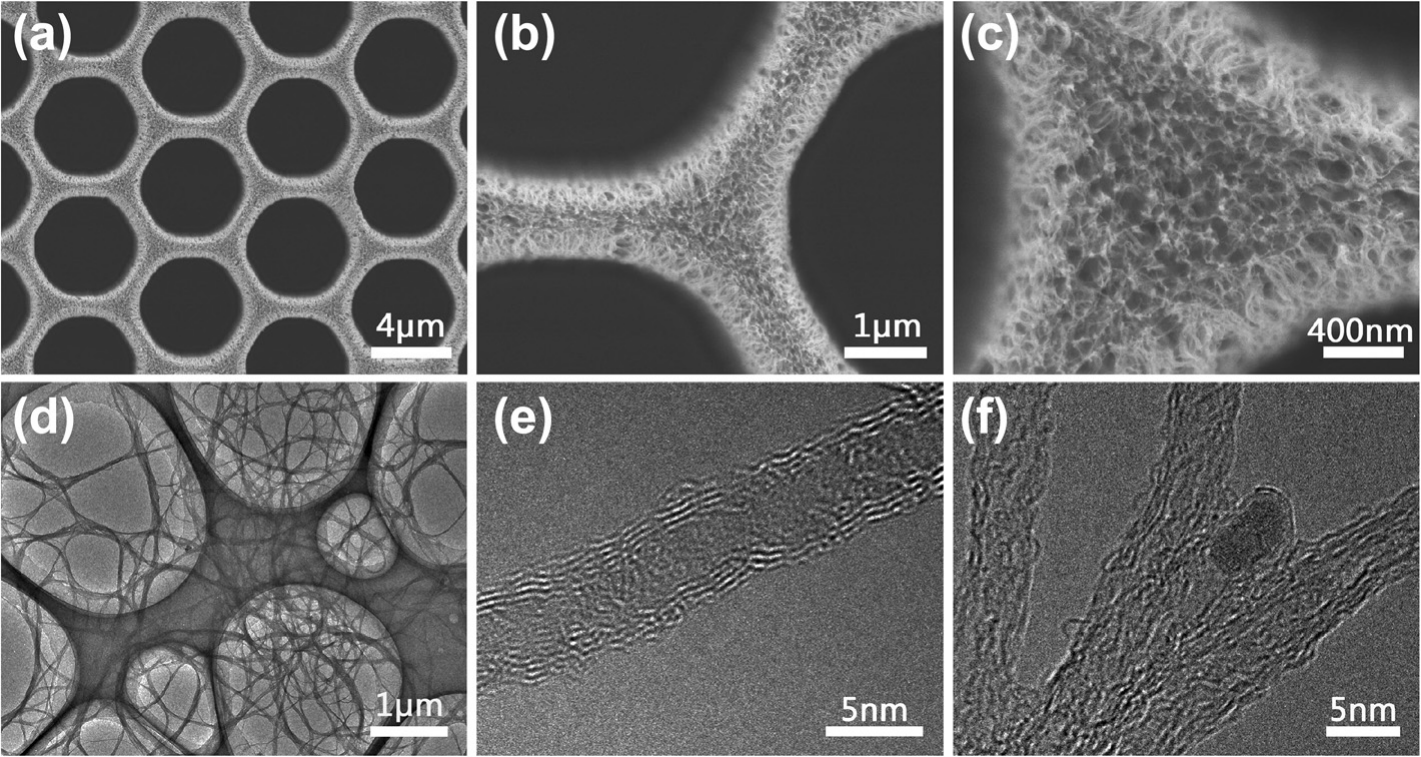
**a**–**c** FESEM images of vertically aligned CNTs grown on the patterned substrate with different magnifications. TEM images of **d** the CNTs. **e** The individual CNT showed multi-walled structure. **f** CNT with catalyst

Figure 2a–c showed the top view FESEM images of as-fabricated CNT distribution on patterned Fe/Al_2_O_3_ thin films with H_2_/C_2_H_2_ = 140/5 sccm. Uniform patterned growth of the CNTs was clearly seen from the low-magnification image (Fig. 2a). The CNTs were well aligned perpendicular to the substrate with very high density as shown in Fig. 2b, c. An observation of Fig. 2c revealed that small quality of CNTs were gathered at the top. And it seemed that the CNTs at the outer edge were a few longer than the center part which may due to the faster growth rate [30]. As the size of catalyst metal could affect the growth rate of CNT [31], the grain size of the catalyst may different in the substrate during the processing procedure. The tubular structure of CNTs was verified by TEM observation (Fig. 2d). The multi-walled CNTs displayed uniform diameters of about 5 nm as shown in Fig. 2e. In general, the carbon precursors are decomposed in high temperature and diffused through the catalyst particles to form a carbon-metal alloy. Then the tubular structures can be formed with the catalysts either at the base or top [32]. Figure 2f showed a view of typical CNT which contained a catalyst particle. The results also indicated that the fabrication of a single CNT was from a catalyst island.

**Fig. 3:**
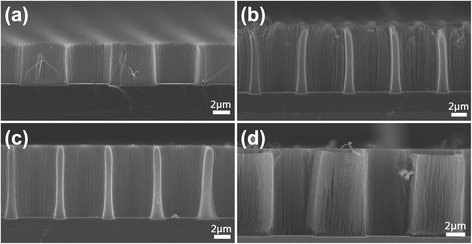
Cross-sectional view of CNTs prepared with different H_2_/C_2_H_2_ mixture ratio. The H_2_/C_2_H_2_ flow rates were **a** 140/2 sccm, **b** 160/5 sccm, **c** 140/5 sccm, and **d** 180/10 sccm, respectively

The CNTs were in alignment on the substrate as shown in the cross-sectional view of FESEM images (Fig. 3). It was obvious that the CNTs grown with different H_2_/C_2_H_2_ mixture ratio changed a lot especially the length. As the ratio that changed from 140/2 to 180/10, the lengths of CNTs were approximately 5.23, 8.8, 9.6, and 12.3 μm, respectively. The CNT growth rate was increased due to more carbon concentration in H_2_ radicals. In general, the H_2_ can control the deposition rate of hydrocarbon and etch the amorphous carbon which may deposit on top of the catalyst so that catalyst activity can be maintained during the CNT growth [33]. The balance of carbon and hydrogen radicals was crucial for CNT growth [34].

**Fig. 4:**
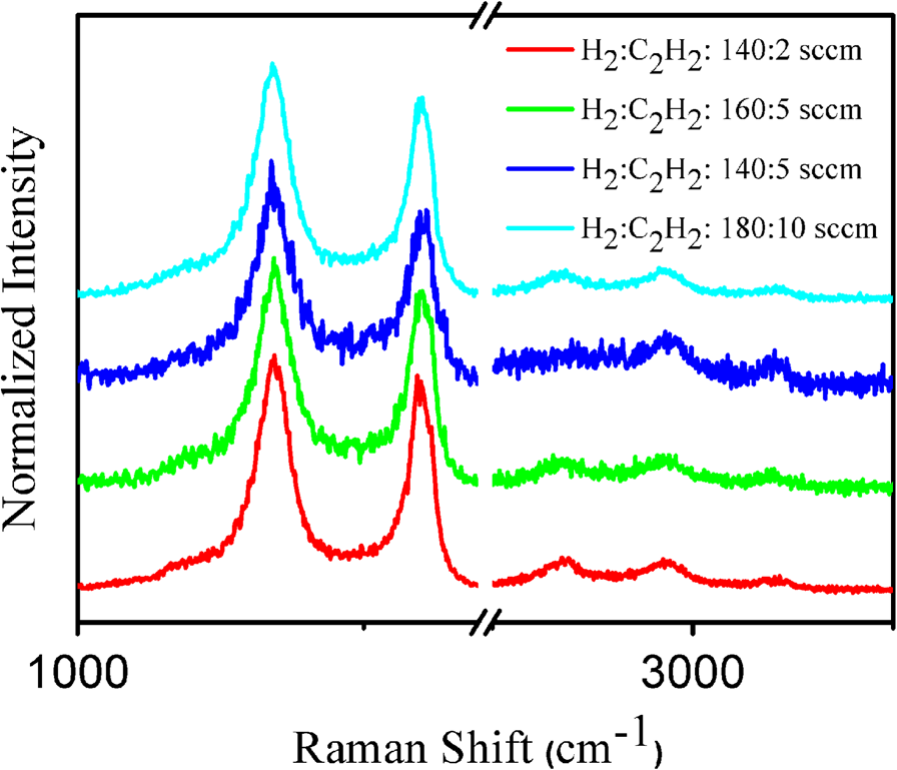
Raman spectra of the vertically aligned CNTs grown on patterned substrates

Raman spectra of the CNTs grown with different H_2_/C_2_H_2_ mixture ratio were shown in Fig. 4. In Raman spectra of carbon materials, the strong band at about 1350–1360 cm^−1^ is attributed to the typical D band assigned to the disorder-induced phonon mode [35]; G band (E_2g_) oriented from the in-plane vibrational mode located at about 1580–1590 cm^−1^ indicating the formation of graphitized structure [36]. In this work, the strong D band at 1344 cm^−1^ indicated the defects and impurities of atoms in CNTs. Herein, the sharp G band with lower intensity relatively to the D band was located at 1600 cm^−1^ which also showed the disorder graphitized structures [37–39]. Seen from Fig. 4, the fraction ratio of the intensity of the D band to G band (I_D_/I_G_) had slightly changed with the H_2_/C_2_H_2_ mixture ratio. Although CNT growth depended on the H_2_/C_2_H_2_ mixture ratio, the microstructure of CNTs did not change a lot. The other three peaks located at 2693 (2D), 2930 (D + G), and 3215 cm^−1^ (2D’) were second-order Raman spectra attributed to combinations of the Raman fundamentals. These bands had also been observed in the Raman spectra of highly ordered pyrolytic graphite [40].

**Fig. 5:**
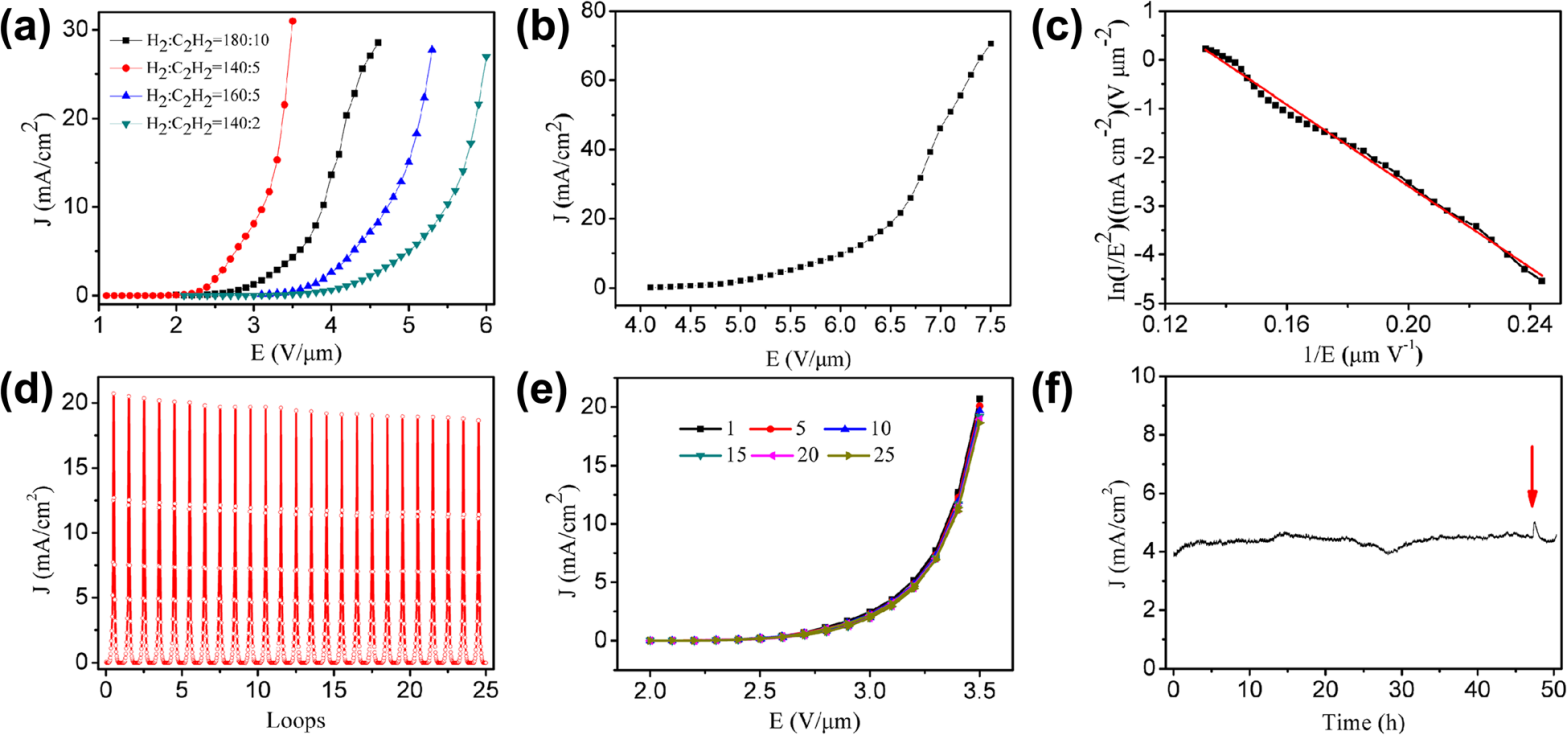
**a** Field emission behaviors of CNTs on patterned substrates grown at different H_2_/C_2_H_2_ mixture ratio. **b** Maximum current density test of the CNTs grown with H_2_/C_2_H_2_ = 140/5 sccm. **c** The F-N plots. **d** The typical curves of the current density (J) varied with the testing time. **e** The J-E curves of the CNT carried out with increased and decreased voltages of different loops. **f** Long-term stability curves of CNT emitters grown H_2_/C_2_H_2_ = 140/5 sccm

The field emission characteristics of the CNTs were investigated shown in Fig. 5. Figure 5a displayed the relationship of emission current density (J) versus applied electric field (E). It seemed that the height of CNT is a factor to influence the field emission characteristics. Seen from Fig. 5a, the field emission properties were increased with the height of the CNTs in a range. However, the CNTs grown with H_2_/C_2_H_2_ = 140/5 sccm exhibited the best compared to the other samples. The turn-on and threshold fields (defined as the electric field required to acquire the current density of 0.1 and 1 mA/cm^2^, respectively) were 2.1 and 2.4 V/μm, respectively. The maximum current density of the CNTs could reach 70 mA/cm^2^ (Fig. 5b) with testing repeatedly which was higher than other reported works [26–28]. Fluctuation and drastic drop did not happen at higher applied voltages which indicated the CNTs had a good contact with the substrate and high-voltage endurance. It could be seen that the CNTs with proper height would benefit to their field emission properties. The field emission characteristics of the CNTs were also analyzed with the Fowler-Nordheim (F-N) theory [41, 42]. The F-N curve shown in Fig. 5c presented a nearly straight line indicated the electron emission was controlled by the tunneling effect. As the ratio that changed from 140/2 to 180/10, the field enhancement factors were 1891, 1966, 2714, and 2759, respectively. The CNTs had lower turn-on field exhibited higher enhancement factor. However, the turn-on field of CNT grown with H_2_/C_2_H_2_ = 140/5 sccm is lower than the CNT grown with 180/10; they had similar enhancement factor. The same phenomenon was also appeared in other works [26, 28]. The field enhancement factor β is strongly dependent on the geometrical shape of the CNTs. More importantly, the local state of the CNTs will also influence the value of β [43].

The emission stability of a field emission electron source is one of the key factors that affect its potential application in vacuum electronic devices. The J-E curves can only show transitory field emission phenomenon in a short time and cannot reflect the field emission behavior sufficiently. Figure 5d showed the looping testing of the CNTs. The anode voltage was increased or decreased by 30 V/step. Seen from loop testing with increased and decreased voltage between 1.5 and 3.5 V μm^−1^ for 25 loops, there is no obvious deterioration of the maximum current density. Before the looping testing with maximum current of about 20 mA/cm^2^, the current density of 10 mA/cm^2^ was also tested with no deterioration. Figure 5e displayed the typical J-E curves for different loops of the loop testing. The current density was relatively stable both in the increased and decreased voltage processes. During the increased or decreased voltage process of the field emission testing, desorption and adsorption of the gas molecules will change the work function of CNT and probably lead to a phenomenon of hysteresis [9, 44]. The hysteresis was unnoticeable in this work indicated that desorption and adsorption may reach an equilibrium state. Furthermore, the long-term test of the sample exhibited good stability for 50 h (Fig. 5f). When ionization vacuum gauge was opened, the emission current increased abruptly during the stability test as shown in the arrow pointed position of Fig. 5f. The conditions in vacuum chamber and the surface state of CNT emitters may change in testing process which resulted in the rising of emission current. All these achievements underlined the potential of CNT emitters in applications.

## Conclusions

In summary, the vertically aligned CNTs were synthesized on patterned substrates by PECVD. The field emission properties of CNTs were optimized with different H_2_/C_2_H_2_ mixture ratios. The CNTs exhibited excellent field emission characteristics with high current density and good emission stability. In order to achieve practically available electron field emitters based on CNTs, we should still focus on the enhancement of electron emission density and the structure design.

## References

[1] Milne WI, Teo KBK, Amaratunga GAJ, Legagneux P, Gangloff L, Schnell JP, Semet V, Thien Binh V, Groening O (2004). Carbon nanotubes as field emission sources. J Mater Chem.

[2] Spindt CA (1968). A thin film field emission cathode. J Appl Phys.

[3] Spindt CA, Brodie I, Humphrey L, Westerberg ER (1976). Physical properties of thin film field emission cathodes with molybdenum cones. J Appl Phys.

[4] Au FCK, Wong KW, Tang YH, Zhang YF, Bello I, Lee ST (1999). Electron field emission from silicon nanowires. Appl Phys Lett.

[5] She JC, Deng SZ, Xu NS, Yao RH, Chen J (2006). Fabrication of vertically aligned Si nanowires and their application in a gated field emission device. Appl Phys Lett.

[6] Chuang FY, Sun CY, Chen TT, Lin IN (1996). Local electron field emission characteristics of pulsed laser deposited diamondlike carbon films. Appl Phys Lett.

[7] Zhu W, Kochanski GP, Jin S (1998). Low-field electron emission from undoped nanostructured diamond. Science.

[8] Li J, Chen JT, Shen BS, Yan XB, Xue QJ (2011). Temperature dependence of the field emission from the few-layer graphene film. Appl Phys Lett.

[9] Chen JT, Li J, Yang J, Yan XB, Tay BK, Xue QJ (2011). The hysteresis phenomenon of the field emission from the graphene film. Appl Phys Lett.

[10] Fan SS, Chapline MG, Franklin NR, Tombler TW, Cassell AM, Dai HJ (1999). Self-oriented regular arrays of carbon nanotubes and their field emission properties. Science.

[11] de Heer WA, Chatelain A, Ugarte D (1995). A carbon nanotube field emission electron source. Science.

[12] Bonard JM, Kindb H, Stöcklic T, Nilssona LO (2001). Field emission from carbon nanotubes: the first five years. Solid State Electron.

[13] Lee J, Jung Y, Song J, Kim JS, Lee GW, Jeong HJ, Jeong Y (2012). High-performance field emission from a carbon nanotube carpet. Carbon.

[14] Lahiri I, Wong J, Zhou Z, Choi W (2012). Ultra-high current density carbon nanotube field emitter structure on three-dimensional micro-channeled copper. Appl Phys Lett.

[15] Sridhar S, Tiwary C, Vinod S, Taha-Tijerina JJ, Sridhar S, Kalaga K, Sirota B, Hart AHC, Ozden S, Sinha RK, Harsh, Vajtai R, Choi W, Korda´s K, Ajayan PM (2014). Field emission with ultralow turn on voltage from metal decorated carbon nanotubes. ACS Nano.

[16] Wey Y, Weng D, Yang Y, Zhang X, Jiang K, Liu L, Fan SS (2006). Efficient fabrication of field electron emitters from the multiwalled carbon nanotube yarns. Appl Phys Lett.

[17] Jang HS, Jeon SK, Nahm SH (2010). Field emission properties from the tip and side of multi-walled carbon nanotube yarns. Carbon.

[18] Jang YT, Choi CH, Ju BK, Ahn JH, Lee YH (2003). Fabrication and characteristics of field emitter using carbon nanotubes directly grown by thermal chemical vapor deposition. Thin Solid Films.

[19] Lee CJ, Kim DW, Lee TJ, Choi YC, Park YS, Lee YH, Choi WB, Lee NS, Park GS, Kim JM (1999). Synthesis of aligned carbon nanotubes using thermal chemical vapor deposition. Chem Phys Lett.

[20] Park D, Kim YH, Lee JK (2003). Synthesis of carbon nanotubes on metallic substrates by a sequential combination of PECVD and thermal CVD. Carbon.

[21] Meyyappan M, Delzeit L, Cassell A, Hash D (2003). Carbon nanotube growth by PECVD: a review. Plasma Sources Sci Technol.

[22] Lee DH, Lee WJ, Kim SO (2009). Highly efficient vertical growth of wall-number-selected, N-doped carbon nanotube arrays. Nano Lett.

[23] Choi WB, Chung DS, Kang JH, Kim HY, Jin YW, Han IT, Lee YH, Jung JE, Lee NS, Park GS, Kim JM (1999). Fully sealed, high-brightness carbon-nanotube field emission display. Appl Phys Lett.

[24] Saito Y, Uemura S (2000). Field emission from carbon nanotubes and its application to electron sources. Carbon.

[25] Chen GH, Wang WL, Peng J, He CS, Deng SZ, Xu NS, Li ZB (2007). Screening effects on field emission from arrays of (5,5) carbon nanotubes: quantum mechanical simulations. Phys Rev.

[26] Shahi M, Gautam S, Shah PV, Jha P, Kumar P, Rawat JS, Chaudhury PK, Harsh, Tandon RP (2013). Effect of purity, edge length, and growth area on field emission of multi-walled carbon nanotube emitter arrays. J Appl Phys.

[27] Huang YJ, Chang HY, Chang HC, Shih YT, Su WJ, Ciou CH, Chen YL, Honda S, Huang YS, Lee KY (2014). Field emission characteristics of vertically aligned carbon nanotubes with honeycomb configuration grown onto glass substrate with titanium coating. Mater Sci Eng B.

[28] Hung YJ, Huang YJ, Chang HC, Lee KY, Lee SL (2014). Patterned growth of carbon nanotubes over vertically aligned silicon nanowire bundles for achieving uniform field emission. Nanoscale Res Lett.

[29] Yun Y, Shanov V, Tu Y, Subramaniam S, Schulz MJ (2006). Growth mechanism of long aligned multiwall carbon nanotube arrays by water-assisted chemical vapor deposition. J Phys Chem B.

[30] Choi YC, Shin YM, Bae DJ, Lim SC, Lee YH, Lee BS (2001). Patterned growth and field emission properties of vertically aligned carbon nanotubes. Diam Relat Mater.

[31] Choi YC, Shin YM, Lee YH, Lee BS, Park GS, Choi WB, Lee NS, Kim JM (2000). Controlling the diameter, growth rate, and density of vertically aligned carbon nanotubes synthesized by microwave plasma-enhanced chemical vapor deposition. Appl Phys Lett.

[32] Merkulov VI, Melechko AV, Guillorn MA, Lowndes DH, Simpson ML (2001). Alignment mechanism of carbon nanofibers produced by plasma-enhanced chemical vapor deposition. Appl Phys Lett.

[33] Honda SI, Katayama M, Lee KY, Ikuno T, Ohkura S, Oura K, Furuta H, Hirao T (2003). Low temperature synthesis of aligned carbon nanotubes by inductively coupled plasma chemical vapor deposition using pure methane. Jpn J Appl Phys.

[34] Zhang G, Mann D, Zhang L, Javey A, Li Y, Yenilmez E, Wang Q, McVittie JP, Nishi Y, Gibbons J, Dai H (2005). Ultra-high-yield growth of vertical single-walled carbon nanotubes: hidden roles of hydrogen and oxygen. Proc Natl Acad Sci.

[35] Nemanich RJ, Solin SA (1979). First- and second-order Raman scattering from finite-size crystals of graphite. Phys Rew B.

[36] Wang Y, Alsmeyer DC, McCreery RL (1990). Raman spectroscopy of carbon materials: structural basis of observed spectra. Chem Mater.

[37] Tuinstra F, Koenig JL (1970). Raman spectrum of graphite. J Chem Phys.

[38] Ferrari AC (2007). Raman spectroscopy of graphene and graphite: disorder, electron–phonon coupling, doping and nonadiabatic effects. Solid State Commun.

[39] Yoshimura A, Yoshimura H, Shin SC, Kobayashi K, Tanimura M, Tachibana M (2012). Atomic force microscopy and Raman spectroscopy study of the early stages of carbon nanowall growth by dc plasma-enhanced chemical vapor deposition. Carbon.

[40] Ni ZH, Fan HM, Feng YP, Shen ZX, Yang BJ, Wu YH (2006). Raman spectroscopic investigation of carbon nanowalls. J Chem Phys.

[41] Sharma H, Agarwal DC, Sharma M, Shukla AK, Avasthi DK, Vankar VD (2013). Tailoring of structural and electron emission properties of CNT walls and graphene layers using high-energy irradiation. J Phys D Appl Phys.

[42] Himani S, Agarwal DC, Sharma M, Shukla AK, Avasthi DK, Vankar VD (2014). Structure-modified stress dynamics and wetting characteristics of carbon nanotubes and multilayer graphene for electron field emission investigations. ACS Appl Mater Interfaces.

[43] Li J, Chen JT, Luo BM, Yan XB, Xue QJ (2012). The improvement of the field emission properties from graphene films: Ti transition layer and annealing process. AIP Adv.

[44] Li C, Fang G, Yang X, Liu N, Liu Y, Zhao X (2008). Effect of adsorbates on field emission from flame-synthesized carbon nanotubes. J Phys D Appl Phys.

